# The efficacy and safety of ultrasonic bone scalpel for removing retrovertebral osteophytes in anterior cervical discectomy and fusion: A retrospective study

**DOI:** 10.1038/s41598-023-50545-y

**Published:** 2024-01-02

**Authors:** Zhi Yao, Shishuang Zhang, Weijun Liu, Mengcheng Wei, Weizhi Fang, Qingbo Li, Lei Cai, Zhengkun Wang, Chuankun Zhou, Yichi Zhou

**Affiliations:** 1 Department of Spine Surgery, Wuhan Fourth Hospital; Puai Hospital, Tongji Medical College Affiliated to Huazhong University of Science and Technology, No. 473 Hanzheng Street, Qiaokou District, Wuhan, 430033 China; 2 Department of Spine Surgery, Wuhan Fourth Hospital, School of Medicine, Jianghan University, No. 473 Hanzheng Street, Qiaokou District, Wuhan, 430033 China; 3 Department of Spine Surgery, Wuhan Fourth Hospital, No. 473 Hanzheng Street, Qiaokou District, Wuhan, 430033 China

**Keywords:** Diseases, Medical research

## Abstract

In this study, we present a novel surgical method that utilizes the ultrasonic bone scalpel (UBS) for the removal of large retrovertebral osteophytes in anterior cervical discectomy and fusion (ACDF) and evaluate its safety and efficacy in comparison to the traditional approach of using high-speed drill (HSD). A total of 56 patients who underwent ACDF for retrovertebral osteophytes were selected. We recorded patients' baseline information, operation time, intraoperative blood loss, complications, JOA and VAS scores, and other relevant data. The mean operation time and the mean intraoperative blood loss in the UBS group were less than those in the HSD group (P < 0.05). Although both groups exhibited considerable improvements in JOA and VAS scores following surgery, there was no statistically significant difference between the two groups (P > 0.05). Additionally, no significant disparities were found in bone graft fusion between the two groups at 6- and 12-months postsurgery. Notably, neither group exhibited complications such as dura tear or spinal cord injury. Our study found that the use of UBS reduced operative time, minimized surgical bleeding, and led to clinical outcomes comparable to HSD in ACDF. This technique offers an effective and safe method of removing large retrovertebral osteophytes.

## Introduction

Anterior cervical discectomy and fusion (ACDF) has been regarded as a classical surgical method for the treatment of cervical spondylosis since its introduction in the 1950s^1^. This method has shown promising postoperative results for patients with cervical spondylotic myelopathy and cervical spondylotic radiculopathy^1,2^. Neurogenic and neurologic symptoms are usually caused by herniated discs and posterior osteophytes that compress the cervical nerve roots or spinal cord^2–5^. Traditionally, the removal of retrovertebral osteophytes involves a slow process using rongeur or high-speed drill (HSD). However, this method can be time-consuming especially when dealing with large osteophytes and poses huge risks of spinal cord injury and dural tears when the osteophyte adheres to the soft tissue behind the vertebral body. To remove large retrovertebral osteophytes, some surgeons prefer to perform anterior cervical corpectomy and fusion (ACCF). However, ACCF has numerous disadvantages, including high cost, substantial trauma, excessive bone loss, vertebral cancellous bone hemorrhage, and graft displacement^5–7^.

As a novel selective bone cutting tool, the ultrasonic bone scalpel (UBS) shows considerable potential for improving the removal of retrovertebral osteophytes. UBS offers multiple advantages, including soft tissue protection, localized bone tissue hemostasis, and precise manipulation^8,9^. The results of several studies suggest that the ultrasonic bone scalpel (UBS) is safe and effective for various spinal surgeries, including primary lumbar decompression, longitudinal ligamentous ossification dome decompression, and thoracic circumferential decompression^10–13^. However, the efficacy and safety of UBS for removing retrovertebral osteophytes remain uncertain due to a lack of large sample studies.

While UBS has various applications in orthopedics, there is limited research on its use in the cervical spine. To date, there is only one study reporting the application of UBS for removing retrovertebral osteophytes and its method resulted in a prolonged procedure^14^, which is inconsistent with our experience. Currently, there is a lack of comparative studies evaluating the efficacy and safety of UBS compared to HSD. In this study, we describe our experience using UBS to remove retrovertebral osteophytes in ACDF for patients with cervical spondylosis and compare its safety and efficacy with HSD.

## Material and methods

### Patients and data

We conducted a retrospective review of patient data from Wuhan Fourth Hospital who underwent cervical surgery between January 2020 and September 2022. This study was approved by the Ethics Committee of Wuhan Fourth Hospital (document number:KY2023-038-01). The inclusion criteria for this study were as follows: (1) diagnosis of cervical spondylotic radiculopathy, cervical spondylotic myelopathy, or mixed cervical spondylosis; (2) presence of retrovertebral osteophytes, with or without disc protrusion; (3) use of surgical tools including HSD, with or without UBS; (4) implementation of the ACDF procedure; and (5) minimum follow-up of 6 months. The exclusion criteria were as follows: (1) calcification of the dura mater, ligamentum flavum and posterior longitudinal ligament; (2) presence of trauma, tumor, infection, or other systemic diseases; and (3) history of previous cervical surgery.

Based on surgical records, patients were categorized into two groups: the UBS group and the HSD group. Patient information, such as sex, age, diagnosis, and surgery duration, was collected from medical records, surgical records, and follow-up records. Prior to surgery, all patients underwent magnetic resonance imaging and computed tomography scans. Typically, patients were evaluated radiologically during each follow-up visit. Nerve function and pain levels were assessed before surgery and at 3 days, 6 months and 12 months after surgery. Symptoms of radiculopathy included cervical arm pain and paraesthesia, while myelopathy symptoms included gait disturbance, difficulty with fine motor skills, paraesthesia, and erectile dysfunction.

### Surgical procedure

The surgical procedures for all cases followed the standard approach of ACDF, and the surgical instruments used included the HSD (Stryker, Kalamazoo, Michigan, USA) and the UBS (XD860A, SMTP Technology Co., Ltd. Beijing, China).

**Figure 1:**
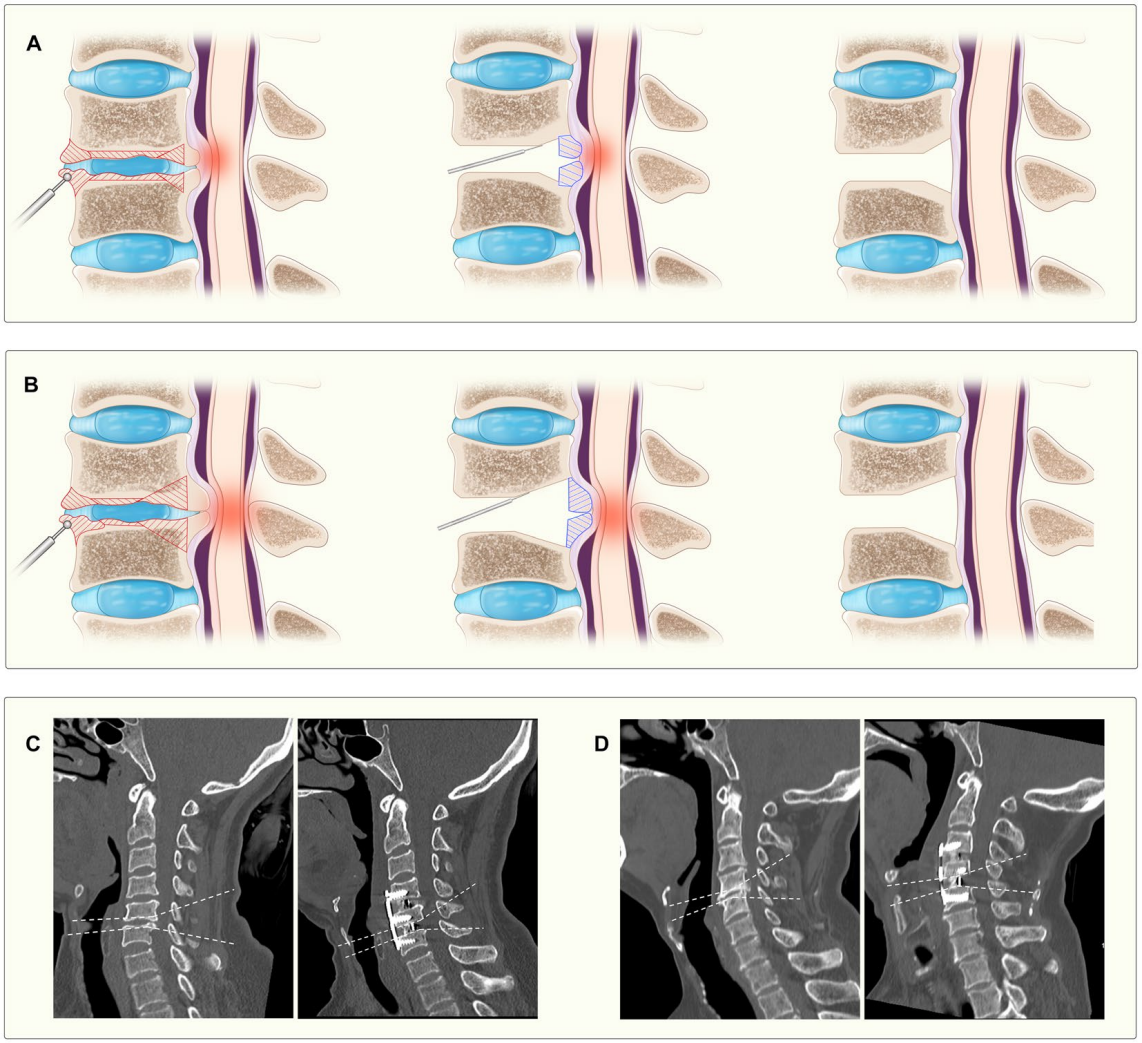
Illustration of surgical procedure using UBS to remove osteophytes and illustrative case presentation. (**A**,**B**) Illustrates different strategies for small and large osteophytes. The red area within the vertebral body represents the region that requires abrasion by the high-speed drill, and the blue area represents the region that are removed with the ultrasonic bone scalpel. Figures (**C,D**) depict preoperative and postoperative assessments of small and large osteophytes, respectively, with the dashed line illustrating the cutting trajectory of the ultrasonic bone scalpel.

**Figure 2:**
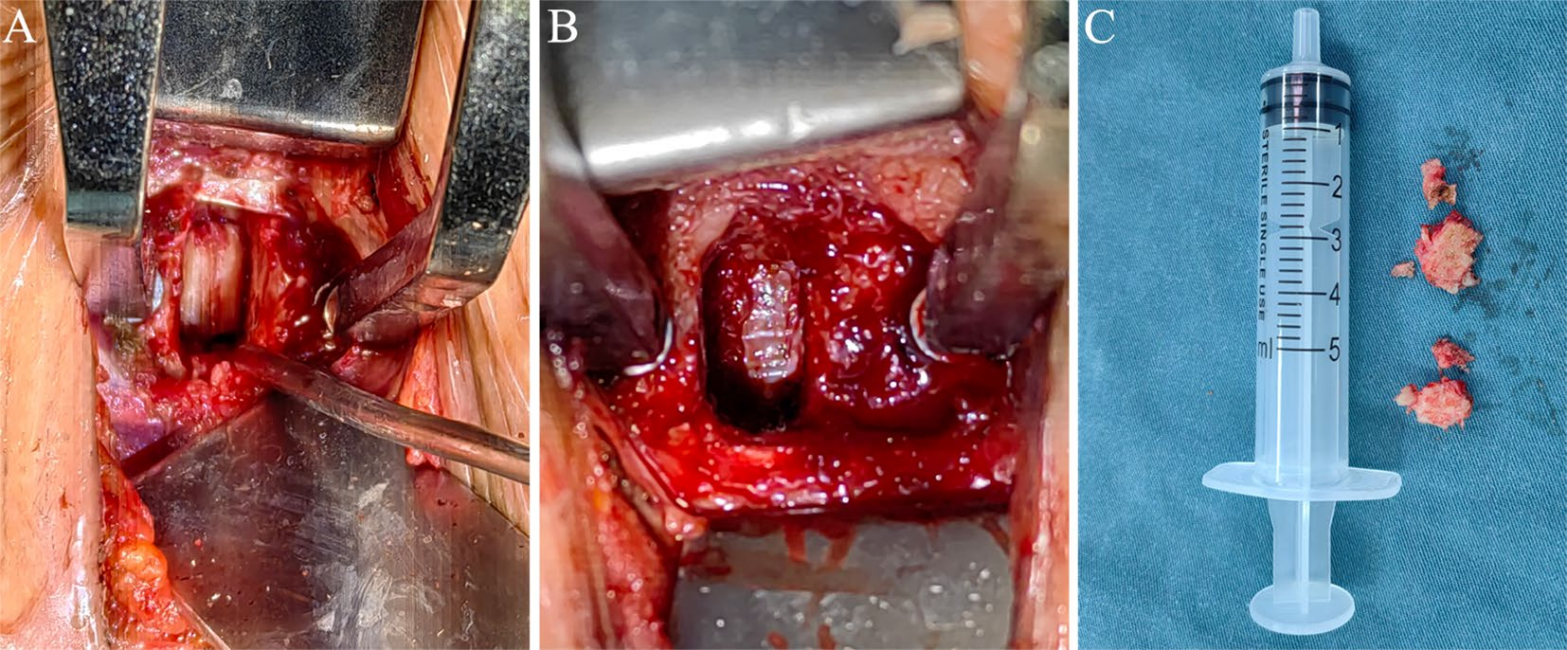
Intraoperative images of osteophytes resection. (**A**) The osteophytes were cut using UBS but have not been removed yet. (**B**) The dura was visible after the complete removal of the osteophytes using the UBS. (**C**) The removed osteophytes.

The patient was placed in the supine position after anesthesia. A transverse incision was made on the right side of the neck, and the platysma muscle was freed and stretched to both sides to expose the cervical vertebrae. Fluoroscopy was used to confirm the correct level of surgery. As shown in Fig. 1, the HSD was first used to remove the anterior cervical osteophytes, to expose as much of the intervertebral disc space as possible, and to create a flat edge of the vertebral body for placement of the titanium plate. The disc was completely removed under the operating microscope, and a portion of the cartilage endplate was removed using the HSD. The depth of drilling was determined by the size of the osteophytes (Fig. 1). When the osteophytes were small, they do not exceed the concavity of the cartilage endplate, the drill was directed 3/4–4/5 into the intervertebral space, and the UBS was used to cut the osteophytes parallel to the endplate. When the osteophytes were large and exceeded the concavity of the cartilage endplate, the drill was directed 1/2–2/3 into the intervertebral space. Subsequently, a small portion of the posterior vertebral body and the medial Luschka joint was removed using the HSD to form a sharp bone groove to facilitate the UBS operation without slippage (Fig. 2). The osteophytes were then completely severed with the UBS along the bone groove. Then, the UBS was used to completely cut the osteophytes obliquely upward or downward. Finally, the osteophytes were carefully removed under the protection of a nerve dissector. If the osteophytes were too large to be removed, they could be cut into pieces by UBS and removed out separately (Fig. 2).

To ensure complete removal of the osteophyte and full relief of compression, a never dissector was used during surgery. Subsequently, a PEEK-cage and allograft bone were implanted, and the upper and lower vertebral bodies of the surgical disc were fixed using screws and titanium plates (Fig. 1).

### Outcome measurements

The level of pain in patients with cervical spondylotic radiculopathy or mixed cervical spondylosis was assessed by the visual analog scale (VAS) score. The Japanese Orthoped Association (JOA) score and improvement rate (IR) were used to evaluate neurological function and symptom improvement in patients with cervical spondylotic myelopathy or mixed cervical spondylosis. Bone graft fusion was evaluated through 6 months and 12 months postoperative X-ray scans or CT scans, with the presence of bone trabeculae between the bone graft and the adjacent endplates being used as an indicator of fusion. Bone graft was considered fused if patients met one of the following conditions^15,16^: (1) CT scan showed the presence of bone trabeculae between the bone graft and the adjacent endplates; (2) plain film showed the presence of bone trabeculae between the bone graft and the adjacent endplates, and the patient did not experience pain during anterior extension and retroflexion of the neck.

### Statistical analysis

The data were analyzed using SPSS 25.0 statistical software and are presented as the means ± standard deviations or numbers. Student’s t test was used to compare quantitative variables with a normal distribution, while the Mann‒Whitney U test was used for quantitative variables with a nonnormal distribution, and the chi-square test was used for qualitative variables when comparing two groups. *P* values < 0.05 were considered statistically significant.

### Ethics appoval and conent to partcipate

The present study was performed under a protocol authorized by the Ethics Committee of the Wuhan Fourth Hospital (Document number:KY2023-038-01) and in accordance with the Declaration of Helsinki (Ethical Principles for Medical Research Involving Human Subjects). All the patients agreed with the data and publication of the manuscript and all patients provided written informed consent.

## Results

As presented in Table 1, a total of 56 patients were enrolled in this study, which comprised 32 males and 24 females with a mean age of 57.8 years (range: 33–76 years). The UBS group consisted of 29 cases, while the HSD group had 27 cases. The baseline demographics and diagnoses in the two groups are summarized in Table 1. No statistically significant difference was observed between the two groups (*P* > 0.05).

**Table 1:** Patient characteristics.

	UBS group	HSD group	*P* value
Age, years	59.0 ± 10.0	56.5 ± 9.5	0.338
Gender, males/females	16:13	16:11	0.757
Diagnosis			0.841
Cervical spondylotic myelopathy	15	14	–
Cervical spondylotic radiculopathy	12	10	–
Mixed cervical spondylosis	2	3	–
Surgery segment			0.725
Single segment	12	9	–
Two segments	13	15	–
Three segments	4	3	–

### Operation-related data

As shown in Table 2, the mean operation times in the UBS group and HSD group were 92.1 ± 17.2 and 123.7 ± 22.2 min, respectively. The operation time in the UBS group was significantly shorter than that in the HSD group (*P* < 0.05). The intraoperative blood loss for patients in the UBS group and HSD group were 71.4 ± 53.2 and 112.0 ± 63.4 mL, respectively. The intraoperative blood loss in the UBS group was significantly lower than that in the HSD group (*P* < 0.05). There were no instances of dural tears or spinal cord injuries in either group.

**Table 2:** Operation-related data.

	UBS group	HSD group	*P* value
Operation time, min	92.1 ± 17.2	123.7 ± 22.2	0.000*
Intraoperative blood loss, mL	71.4 ± 53.2	112.0 ± 63.4	0.007*
Dural tear	0	0	–
Spinal cord injury	0	0	–

*P < 0.05.

### Clinical outcomes

As shown in Table 3, the preoperative JOA scores were 10.4 ± 0.6 and 10.3 ± 0.6 points in the UBS and HSD groups, respectively (*P* > 0.05). Six months after the surgery, the JOA scores of both groups significantly increased (*P* < 0.05), but no significant difference was observed between the two groups (*P* > 0.05). The IR showed no significant difference as well (*P* > 0.05). Additionally, there were no significant differences in the preoperative and postoperative VAS scores (*P* > 0.05).

**Table 3:** Clinical outcomes.

	UBS group	HSD group	*P* value
JOA scores, preoperative	10.4 ± 0.6	10.3 ± 0.6	0.984
JOA scores, 6 months postoperative	14.3 ± 0.5	14.3 ± 0.8	0.772
IR (%)	59.3 ± 6.2	59.8 ± 10.1	0.815
VAS scores, preoperative	6.3 ± 0.5	6.0 ± 0.6	0.182
VAS scores, 6 months postoperative	2.2 ± 0.4	2.2 ± 0.4	0.692
Bone graft fusion, 6 months postoperative (%)	93.1	88.9	0.580
Bone graft fusion, 12 months postoperative (%)	100	94.7	0.460

Six months after surgery, 93.1% and 88.9% of patients in the UBS and HSD groups, respectively, achieved bone graft fusion. Furthermore, twelve months after surgery, 100% and 94.7% of patients in the UBS and HSD groups, respectively, achieved bone graft fusion. There was no significant difference between the two groups at either 6 months or 12 months after surgery (*P* > 0.05).

## Discussion

In this study, we present a novel surgical technique that uses the UBS to effectively and safely remove large retrovertebral cervical osteophytes during ACDF. Furthermore, we investigated the safety and efficacy of the UBS by classifying patients with cervical spondylosis and large posterior cervical osteophytes into two groups: the UBS and HSD groups. Our results demonstrate that the use of the UBS resulted in shorter operation times, decreased intraoperative blood loss and comparable clinical outcomes compared to the HSD group. The clinical outcomes of this study were consistent with previous research both groups showed considerable improvements in JOA and VAS scores postsurgery, and there was no significant difference between them. Moreover, there was no significant difference in bone graft fusion between the groups.

Regarding the removal of retrovertebral osteophytes, HSD can completely abrade them away. However, to prevent the occurrence of dural tears and spinal cord injuries, this step needs much time to complete, especially when the posterior osteophyte is large or near the dura. In addition, the use of HSD can result in several shortcomings, including direct heat injury to surrounding tissues and the need for frequent irrigation and suction during the procedure^17^. Regarding UBS, Grauvogel et al. conducted a study using an angled UBS to remove posterior cervical osteophytes, however, they reported prolonged operative time^14^.

To achieve complete, safe, and efficient removal of osteophytes, our team developed a novel surgical technique that utilizes the selective bone cutting tool of the UBS. In our study, HSD was used only to remove the anterior part of the large retrovertebral osteophytes. Then, we used the UBS to cut the large retrovertebral osteophytes and subsequently removed the entire block of the remaining retrovertebral osteophytes. Our method has four main advantages. First, our method is safer than using HSD alone, as it reduces contact with the dura and spinal cord and exploits the characteristics of tissue selectivity. This characteristic of the UBS cutting bone tissue while protecting soft tissue allows surgeons to safely remove retrovertebral osteophytes without the risk of dural tears and spinal cord injury. Second, our method saves surgical time as we remove the entire block of the remaining retrovertebral osteophytes instead of grinding it down bit by bit with HSD. Third, the UBS can remove large osteophytes under blind visualization. Due to the potential damage to surrounding tissues, HSD requires osteophyte removal under direct visualization. Due to the limited height of the intervertebral space, removing more endplates and cancellous bone is necessary to achieve direct visualization. On the other hand, the UBS, thanks to its protective characteristic toward soft tissues, can remove osteophytes without the need for direct visualization. Finally, the UBS has longer and thinner blades then the HSD, facilitating the surgical procedure.

Notably, the choice of the blade is also very important. Our technique differs from the use of an angled ultrasonic bone knife as reported by Grauvogel et al.^14^. We believe that without partial resection of the vertebral body, the surgeon's field of view would be limited, and the operation time and risk of dural tear and spinal cord injury would increase. Liu et al. reported the use of a spoon-shaped blade in ACDF^18,19^, but we believe that removing osteophytes bit by bit under direct visualization with this blade may result in suboptimal efficiency. Moreover, using a spoon-shaped blade to remove osteophytes may cause tension on the dura mater, which could be a contributing factor to the higher incidence of dural membrane injury observed in their study.

In our study, we first compared the clinical characteristics of the use of the UBS for the removal of large cervical retrovertebral osteophytes to that of the traditional HSD. Consistent with previous studies, the use of UBS can save operation time^11,12,20–27^. Several reasons contribute to the reduction in operation time, aside from the method used in our study of removing the entire posterior osteophytes. First, traditional tools used for laminectomy or corpectomy often lead to substantial blood loss from cancellous bone. Using the UBS can minimize bleeding and shorten the time required for hemostasis. Second, traditional tools such as HSD and laminectomy rongeurs require surgeons to exercise caution while dealing with osteophytes to prevent dural tears and spinal cord injury, which could lead to a longer operation time. Notably, a prior study that used UBS to remove retrovertebral osteophytes reported increased operation time, but no comparison was made with HSD^14^. The authors proposed that this could be attributed to the surgeon's insufficient familiarity with UBS, inconvenient handling, and inadequate documentation of the intraoperative use for scientific purposes^14^.

Our study demonstrated that using the UBS significantly reduces intraoperative blood loss, which is consistent with previous research^26–28^. The cavitation effect of using the UBS, allowing for local hemostasis, and its tissue selectivity, which reduces vascular damage are the reasons for this advantage. However, it is worth noting that a study on the treatment of thoracic ossification of the posterior longitudinal ligament with circumferential decompression showed that the UBS does not offer any advantage in reducing blood loss when vascular injury cannot be avoided^29^. This finding further emphasizes the fact that the UBS mainly reduces intraoperative blood loss by reducing bone bleeding. Moreover, the aforementioned time-saving advantage is also critical for reducing the amount of intraoperative blood loss.

Regarding postoperative complications, our study found no instances of dural tears or spinal cord injuries in either the UBS or HSD groups. However, it should be noted that previous studies have reported cases of UBS-induced dural tears and spinal cord injuries, highlighting the need for caution^10,21,30,31^. Heat injury is a potential risk to be vigilant about, although using the UBS carries a lower risk of heat injury compared to HSD due to its water-cooling system. Prolonged local retention of the UBS can still cause overheating, leading to bone necrosis, dura tear, and spinal cord injury. Chen et al. recommended that the UBS tip should not remain on one point for more than 5 s^21^. In our experience, moving the UBS layer by layer continuously can minimize the risk of overheating and reduce the risk of complications. Moreover, in cases of dural calcification, preoperative CT will show a prominent double-track sign, indicating that UBS should be used with caution, as it may easily cause a dural tear^20,22,30^. In such cases, the posterior approach may be a more appropriate choice.

Our study has some limitations that need to be acknowledged when interpreting the findings. First, it is a retrospective comparative study and not a randomized controlled trial, which could lead to selection bias and confounding factors. Second, the data were collected from one surgeon at a single medical institution, which could restrict the generalizability of the results. Therefore, future studies involving multiple surgeons from various centers are necessary to validate our findings. Finally, the sample size of this study was relatively small, and larger studies are required to further investigate the safety and efficacy of the UBS in cervical spine surgery.

## Conclusion

In conclusion, our study indicates that the use of the UBS for posterior cervical osteophyte removal is a safe and effective method that provides satisfactory outcomes comparable to traditional instruments. In addition, this method is associated with reduced operative time and intraoperative blood loss. Therefore, combining UBS with HSD-assisted ACDF appears to be a superior option for removing large posterior cervical osteophytes.

## Data Availability

The datasets generated and analyzed during the current study are not publicly available due to patient privacy protection but are available from the corresponding author on reasonable request.

## Abbreviations

HSD: High-speed drill
ACDF: Anterior cervical discectomy and fusion
UBS: Ultrasonic bone scalpel
ACCF: Anterior cervical corpectomy and fusion
OPLL: Ossification of posterior longitudinal ligament
